# Editorial: Cancer of gastrointestinal tract: Novel insight into the molecular mechanisms related to inflammation and therapeutic targets

**DOI:** 10.3389/fphar.2022.970491

**Published:** 2022-08-30

**Authors:** Aditi Banerjee, Laishram Pradeepkumar Singh, Tamaki Ikuse

**Affiliations:** ^1^ Department of Pediatrics, University of Maryland School of Medicine, Baltimore, MD, United States; ^2^ Department of Zoology, Kalyani University, Kalyani, West Bengal, India; ^3^ Department of Pediatric and Adolescent Medicine, Juntendo University Graduate School of Medicine, Tokyo, Japan

**Keywords:** inflammation, gastric cancer, therapeutic target, molecular mechanism, cytokines

Prolonged gastric Inflammation due to an elevated number of pathogens (virus bacteria, etc.) in the intestinal epithelium leads to severe pathological ailments, including gastrointestinal mucositis, gastric cancer, and colon cancer, etc. (Shiu et al., 2015; Banerjee et al., 2016; Sharda et al., 2021). Cellular and tissue damage caused by the upregulated Matrix metalloproteinases are associated with the pathogenesis of gastric inflammation including gastric ulcers (Swarnakar et al., 2005; Singh et al., 2011). The pathological symptoms of such conditions are altered mucosal architecture, vessel tortuosity, enlargements, and leakage (Fornasarig et al., 2021). In addition, inflammation accompanies increased microvascular permeability that is induced by crucial regulators such as inflammatory mediators. The leukocyte interacts with the vascular endothelium often in concert with the soluble inflammatory mediators released from the damaged tissues, which further induces the release of proteolytic enzymes and free radicals. A series of molecular and cellular changes prompt an angiogenic response with a temporary disruption of the vascular barrier, extravasation of plasma, and fibrin deposition. The orchestration of enzymatic machinery post-inflammatory response provides an ideal situation for the invasion of leukocytes through tissue barriers such as the basement membrane and interstitial matrix, and furthers the provisional fibrin matrix deposition in response to increased vascular permeability. Moreover, Unfolded protein response (UPR) mediated Endoplasmic Reticulum (ER) stress is responsible for gastrointestinal tract inflammation and is regarded as a potential initiating factor in intestinal inflammation. It is well known that both genetic and environmental factors drive ER stress in the intestinal epithelium and consequently result in inflammation. Therefore, angiogenic signals and UPR-mediated ER stress are consequent events of gastric inflammation including cancers. The molecular mechanisms and the pathways that regulate such activities are not clearly understood.

This Research Topic on “*Cancer of Gastrointestinal Tract: Novel Insight into the Molecular Mechanisms Related to Inflammation and Therapeutic Targets*,” examines unresolved issues in the settings and combinations outlined below.

1) Esophageal Cancer (EC), which is one of the most aggressive human cancers with poor prognosis, and the overall 5-year survival rate is less than 20 percent (Zhang, 2013; Domper Arnal et al., 2015). Hassan et al. explored this issue and found that aggressive esophageal cancer cells are associated with elevated c-Myc expression. Using a xenograft study, the authors demonstrated that lavopiridol monotherapy or combination therapies exhibited significant survival benefits in mice. The authors concluded that aggressive esophageal cancer cells with elevated c-Myc expression are an effective therapeutic target for the CDK inhibitor flavopiridol either alone or in combination with other cytotoxic or targeted agents .
2) Colorectal Cancer (CRC) is one of the most common gastrointestinal malignancies, with high morbidity and mortality rates. Several biomarkers are available for the prognosis of the patient outcome in CRCs. In this Research Topic, Koshino et al. and their research group identified two biomarkers, PDZ-binding kinase (PBK) and phospho-histone H3 (PHH3) and provided their inverse association for colon cancer progression. Moreover, the authors also identified PBK-mediated suppression of the migration and invasion of CRC cells as one of the potential mechanisms by which higher expression of these cellular proliferation-associated proteins provides better survival for CRC patients. This study also demonstrated that PBK could be a novel candidate for targeted therapeutics for the treatment of PBK positive CRC patients
3) Chemotherapy-induced gastrointestinal toxicities are associated with inflammation and damage to gastric mucosa resulting in pain, nausea, vomiting, bloating, constipation, diarrhea, and ulceration (Eng, 2009). About 80% of the patients that received a standard dose of irinotecan CPT11, a topoisomerase I (TOP1) inhibitor, and exhibit diarrhea-associated intestinal mucositis (Gelibter et al., 2019). Yue et al. showed the berberine’s potential efficacy and underlying mechanisms behind the mitigation of CPT11-induced mucositis. The bacterial β-glucuronidase (GUS), an enzyme expressed by intestinal microbiota, converts the inactive CPT11 metabolite SN38G to the active metabolite SN38 to induce intestinal mucositis. For the first time, the group identified the remission effects of berberine on intestinal mucositis induced by CPT11 without impairing the anti-colorectal cancer efficacy of CPT11 partially through inhibition of bacterial GUS enzyme .
4) Poloznikov et al. investigated the impact of combinations of standard-of-care therapy and 9-ING-41, a small molecule inhibitor of GSK-3β, on colon cancer using cell lines and patient-derived tumor organoid models. Notably, substantial similarities in the changes in the transcriptomic profile were observed after the inhibition of GSK-3β and the suppression of STK33, which provided an interesting point for future research .
5) The study by Wu et al aimed to determine whether serum uric acid is related to the survival time of patients with hepatocellular carcinoma (HCC) and whether the inhibition of uric acid production affects the progression and survival of DEN (diethylnitrosamine)-induced HCC rats. Their results demonstrated that the increased serum uric acid level is a prognostic factor for the poor survival in advanced HCC patients, and purine metabolism was significantly altered in such patients. In addition, febuxostat intervention of xanthine oxidase (XOD) enzyme, significantly delayed liver cancer progression and prolonged the survival time of DEN-induced HCC rats by reducing reactive oxygen species (ROS) production .
6) Rahman et al. reviewed existing standard molecular treatment strategies by focusing on the relationship between chemotherapy and autophagy-mediated gastric cancer (GC) management. GC is the second leading cause of cancer-associated mortality globally, in which malignant cells develop in the lining of the stomach, resulting in indigestion, pain, and stomach discomfort. The authors delineated that targeting autophagy by small-molecule activators or inhibitors (single or in combination) and the use of phytochemicals can provide novel therapeutic approaches for GC management .
7) *Helicobacter pylori* (*H. pylori*) is a Gram-negative bacterium that colonizes the human stomach and causes chronic gastritis, peptic ulcers, and gastric adenocarcinoma. The *H. pylori* TNFα inducing protein (Tipɑ) is a virulence factor that has been shown to induce multiple pro-inflammatory cytokines in addition to TNFα *in vitro*. In the article by Wright, et al. the group elucidates the role of Tipα in promoting inflammation using an *in vivo* model. They also identify the molecular pathways associated with Tipα associated virulence. Furthermore, microarray analyses of gastric epithelial cell cultures treated with purified Tipα protein demonstrated upregulation of the NFκB pathway. This report provides *in vivo* evidence indicating that Tipα contributes to *H. pylori* pathogenesis and may promote precancerous events that put the host at risk for developing gastric cancer .
